# *Myrcia splendens* (Sw.) DC. (syn. *M. fallax* (Rich.) DC.) (Myrtaceae) Essential Oil from Amazonian Ecuador: A Chemical Characterization and Bioactivity Profile

**DOI:** 10.3390/molecules22071163

**Published:** 2017-07-12

**Authors:** Laura Scalvenzi, Alessandro Grandini, Antonella Spagnoletti, Massimo Tacchini, David Neill, José Luis Ballesteros, Gianni Sacchetti, Alessandra Guerrini

**Affiliations:** 1 Department of Earth Science, Universidad Estatal Amazónica, Puyo 160106, Ecuador; 2 Department of Life Sciences and Biotechnology (SVeB), UR7 Terra&Acqua Tech, University of Ferrara, Ferrara 44121, Italy; alessandro.grandini@unife.it (A.G.); antonella.spagnoletti@unife.it (A.S.); massimo.tacchini@unife.it (M.T.); gianni.sacchetti@unife.it (G.S.); 3 Department of Life Science, Universidad Estatal Amazónica, Puyo 160106, Ecuador; dneill@uea.edu.ec; 4 Department of Life Sciences, Universidad Politécnica Salesiana, Quito 170525, Ecuador; jballesterosl@ups.edu.ec

**Keywords:** *Myrcia splendens*, essential oil, cytotoxic activity, antibacterial activity, antioxidant activity, bioautographic assay

## Abstract

In this study, we performed the chemical characterization of *Myrcia splendens* (Sw.) DC. (Myrtaceae) essential oil from Amazonian Ecuador and the assessment of its bioactivity in terms of cytotoxic, antibacterial, and antioxidant activity as starting point for possible applicative uses. *M. splendens* essential oil, obtained by hydro-distillation, was analyzed by Gas Chromatography-Mass Spectrometry (GC-MS) and Gas Chromatography-Flame Ionization Detector (GC-FID): the major components were found to be *trans*-nerolidol (67.81%) and α-bisabolol (17.51%). Furthermore, we assessed the cytotoxic activity against MCF-7 (breast), A549 (lung) human tumor cell lines, and HaCaT (human keratinocytes) non-tumor cell line through 3-(4,5-dimethyl-2-thiazolyl)-2,5-diphenyl-2-*H*-tetrazolium bromide (MTT) test: promising results in terms of selectivity and efficacy against the MCF-7 cell line (IC_50_ of 5.59 ± 0.13 μg/mL at 48 h) were obtained, mainly due to α-bisabolol. Furthermore, antibacterial activity against Gram positive and negative bacteria were performed through High Performance Thin Layer Chromatography (HPTLC) bioautographic assay and microdilution method: *trans*-nerolidol and β-cedren-9-one were the main molecules responsible for the low antibacterial effects against human pathogens. Nevertheless, interesting values of Minimum Inhibitory Concentration (MIC) were noticeable against phytopathogen strains. Radical scavenging activity performed by HPTLC bioautographic and spectrophotometric 1,1-diphenyl-2-picrylhydrazyl (DPPH) approaches were negligible. In conclusion, the essential oil revealed a good potential for plant defense and anti-cancer applications.

## 1. Introduction

Essential oils are mixtures of complex and fragrant substances, employed in many applicative fields, from aromatherapy to medicine and pharmacy, as well as the agro-food industry and phytoiatry [1]. The applicative importance of essential oils in such diversified fields relies mainly on their chemical characterization as mixtures of low molecular weight lipophilic substances and on their biological properties often due to synergistic effects of two or more constituents [2]. In fact, many research papers about essential oils focus on their chemical portrait and in vitro biological properties in terms of antioxidant, antimicrobial, and cytotoxic activities with the aim of detecting the most performance. One of the most interesting chemical aspects, emerging from all these researches, is their high chemodiversity, i.e., different composition, sometimes even dramatic, among phytocomplex from plants belonging to the same species, but grown in different geographical conditions and biodiversity contexts. This aspect is more evident for those species from areas characterized by high biodiversity, where the interaction among a higher number of different species results in a propulsive tool for secondary metabolites which give rise to remarkable molecular diversity and abundance in plants [3,4]. Biological activities of essential oils, instead, independently of the methodological target, are particularly focused on the detection of the compounds responsible of the activity and on the detection and quantification of possible synergistic evidences [2]. Among several kinds of biological activities, cytotoxic and antioxidant are the most interesting since the results could lead to deepened research about chemical compounds and/or chemical platforms for new pharmaceutical treatments of inflammation-related diseases, such as cancers [5]. Given all these premises, a chemical and bioactivity study has been performed on the essential oil of *Myrcia splendens* (Sw.) DC. (syn. *M. fallax* (Rich.) DC.) (Myrtaceae) obtained from plants grown in Amazonian Ecuador. Many neotropical Myrtaceae species are known for their traditional health uses, but some of them are scarcely investigated under the chemical point of view and as source of bioactive compounds. Fresh aerial parts of species belonging to *Myrcia* genus are employed in South American traditional medicine for the treatment of diabetes, hypertension, diarrhea, hemorrhage as infusion, and as a compress to treat inflammation and skin infections [6,7]. *Myrcia splendens* is a tree found in neotropic regions, distributed from Eastern Mexico to the South-Eastern Brazilian coastal forests. Crude drugs, derivatives, and preparations from Peruvian [8] and Brazilian plants have been studied and interesting cytotoxic activities were evidenced [9]. Since the Amazonian basin is one of the most important biodiversity hotspots world-wide, a first report on chemical characterization and biological properties of the essential oil of *M. splendens* from Ecuador was performed, with the aim to compare our data with those already reported by related literature.

## 2. Results

### 2.1. Chemical Composition of Essential Oil

The distillation of *M. splendens* leaves gave a colorless essential oil with a yield of 0.11% (*w*/*v*), which was lower than previously reported (0.35%) [10], and its density was 0.906 g/mL. The *M. splendens* essential oil composition is shown in Table 1.

Twenty-two compounds were characterized, corresponding to 97.84% of the total. Sesquiterpenes were predominant and particularly oxygenated sesquiterpenes, corresponding to 85.47% of the total. Monoterpenes, α- and β-pinene, were present in a lower amount (2.19% as total). Among the sesquiterpenes the most abundant were *trans*-nerolidol (67.81%), α-bisabolol (17.51%) and β-caryophyllene (4.21%). The predominance of sesquiterpenes is peculiar to the *Myrcia* genus [12]. *M. splendens* (syn. *M. fallax*) essential oil exhibited a different composition compared to related studies on the same species. In fact, Henriques et al. [10] found α-bisabolol (83%) as the main compound, while Nakamura et al. [13] found it to be α-bisabolene (80%); Alarcón et al. [7] stated that guaiol (31%) and carotol (9.9%) were the most abundant constituents. β-elemene was found as the main compound in the study of Lima et al. [14] and α-pinene characterized the essential oil obtained by Pereira et al. [15]. *trans*-nerolidol was the main compound (80.8%) of *M. bracteata* essential oil [16]. To the best of our knowledge, *trans*-nerolidol was found only in the *M. splendens* species of the present study. β-caryophyllene was already detected in *M. glabra*, *M. multiflora*, *M. cuprea,* and *M. tomentosa* [17] and α-pinene in *M. bombycina* and *M. myrtifolia* [18]. A deep inter- and intraspecific diversity in chemical composition of Myrtaceae essential oils has been reported by various authors. Data obtained on *M. splendens* essential oil from Ecuador seem to confirm this hypothesis. Previous studies suggested that intraspecific diversity of Myrtaceae terpene profiles could be a response of adaptation to a wide range of environmental conditions, in particular the monoterpene: sesquiterpene ratio seems to be influenced by the efficiency of terpene synthases enzymes [19]. Obtained results indicate that endogenous factors, as high genetic diversity, have more influence on the volatile oil variability of Myrtaceae family than environmental aspects [18]. In particular, the differences observed in chemical composition of *M. splendens* essential oils could also depend on Amazonian origin of the studied samples. In effect, the high biodiversity of Amazonian region induces plant secondary metabolism to biosynthetic pathways characterized by diversified chemical profile [3].

### 2.2. Cytotoxic Activity

**Figure 1:**
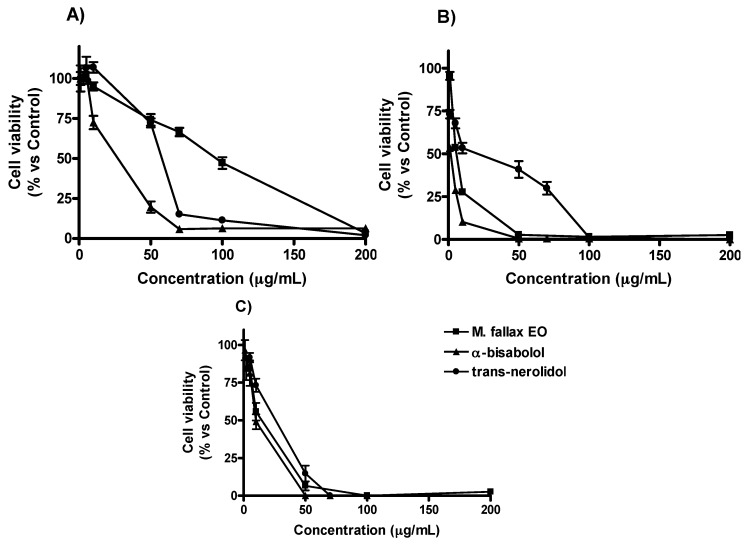
Effects of *M. splendens* essential oil (EO), α-bisabolol and *trans*-nerolidol on viability of A549 (**A**), MCF-7 (**B**) and HaCaT (**C**) cell lines. Cytotoxicity was assessed by MTT test after 48 h.

MTT assay was used to evaluate the effect of *M. splendens* essential oil and its main compounds, α-bisabolol and *trans*-nerolidol, on the cell viability of A549 (human lung cancer), MCF-7 (human breast adenocarcinoma) tumor lines and HaCaT (human keratinocytes) normal cells, as the reference to assess overall cytotoxic activity. All results showed (Figure 1) a dose-dependent inhibition of cell growth and, in particular, α-bisabolol was the most active component against the three studied cell lines, in the dilutions ranging from 1 to 200 µg/mL.

Moreover, at the concentration of 10 µg/mL, α-bisabolol decreased the viability of A549, MCF-7, and HaCaT cell lines of 70%, 10% and 50%, respectively, in comparison with the negative control. The results highlighted that inhibition on cell growth was highly dependent on cell type with IC_50_ values ranging from 1.24 ± 0.03 to 100.99 ± 2.32 μg/mL. In particular, MCF-7 cancer cells showed more growth inhibition than HaCaT cells after 48 h of treatment with α-bisabolol (IC_50_ = 1.24 ± 0.03 μg/mL vs. IC_50_ = 10.15 ± 0.35 μg/mL) and essential oil (IC_50_ = 5.59 ± 0.13 μg/mL vs. IC_50_ = 21.58 ± 1.26 μg/mL) (Table 2). As reported in other studies, IC_50_ values lower than 30 μg/mL present a good chemopreventive potential for the essential oil [20]. However, the HaCaT cells showed to be more sensitive, with IC_50_ values ranging from 10.15 ± 0.35 to 27.76 ± 2.76 μg/mL, than the A549 cell line, with IC_50_ ranging from 54.28 ± 2.39 to 100.99 ± 2.32 μg/mL. Therefore, the evaluation of the cytotoxic activity revealed promising results in terms of selectivity and efficacy of *M. splendens* essential oil against the MCF-7 cell line rather than against the A549 (Table 2).

According to these results, *M. splendens* essential oil may be considered a promissory product to be used for innovative therapeutic or preventive strategies against breast carcinoma. Its cytotoxic activity may depend on the presence of α-bisabolol, which showed a very interesting IC_50_ value after 48 h (1.24 ± 0.03 µg/mL). Previous studies on α-bisabolol exhibited cytotoxic activity against glioma, pancreatic, ovarian, and kidney carcinoma cells [9,21,22]. Furthermore, it was reported as a chemopreventive agent in rat mammary carcinogenesis [23]. Thus the high content of α-bisabolol (17.51%) in the *M. splendens* essential oil could explain the cytotoxic activity against MCF-7 cells. On the other hand, data obtained on A549 were less interesting because the IC_50_ value (27.63 ± 2.01 μg/mL) was very close to 30 μg/mL, the concentration considered as the upper limit for potential chemopreventive activity. In the present study, *trans*-nerolidol exhibited a cytotoxic activity less interesting than α-bisabolol, nevertheless on A549 cells showed IC_50_ value at 48 h (54.28 ± 2.39 μg/mL) in line with the one recorded by Sylvestre et al. [24] (66 ± 12 μg/mL). Antineoplastic activity was also recorded on bowel by a mixture of *cis*- and *trans*-nerolidol [25], suggesting that deeper studies could be carried out in order to assess cytotoxic activity against other kinds of cancer cell lines.

### 2.3. Antibacterial Activity: HPTLC-Bioautography and MIC

Antibacterial properties of *M. splendens* essential oil was assessed against Gram positive and negative bacteria and MIC (Minimum Inhibitory Concentration) was determined by serial microdilution method. *M. splendens* essential oil exhibited a weak antimicrobial activity compared to chloramphenicol, used as a positive control, against all tested bacteria (Table 3).

**Figure 2:**
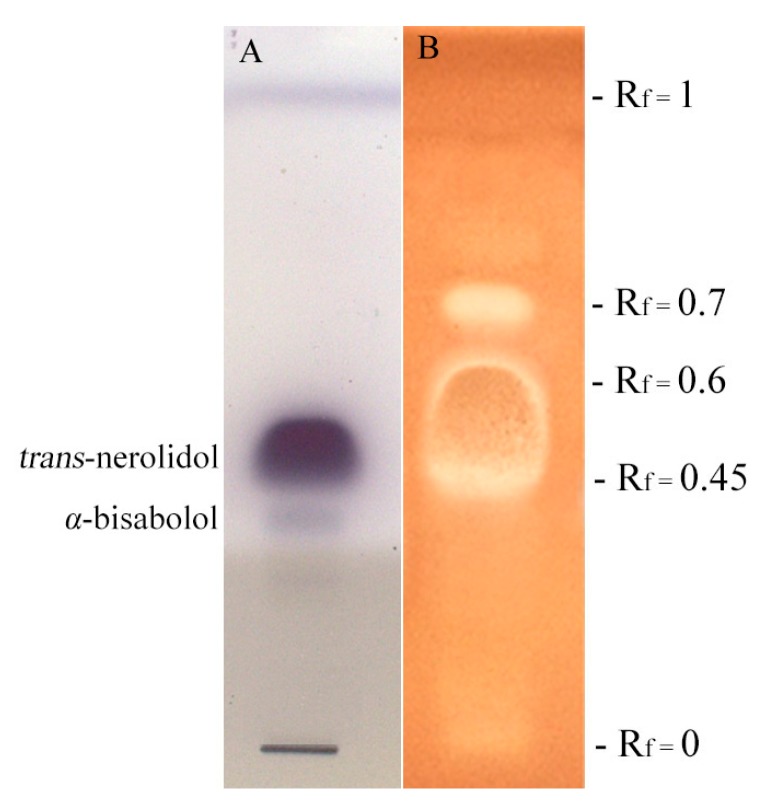
HPTLC (High Performance Thin Layer Chromatography) bioautographic assay performed for antibacterial activity on *S. aureus*, of *M. splendens* essential oil. R_f_: retention factor: it represents the ratio between the migration distance of a substance and the migration distance of the solvent front. R_f_ composition is reported in Table 1. (**A**) HPTLC derivatized with vanillin-phosphoric acid reagent. (**B**) HPTLC performed for antibacterial activity on *S. aureus.*

HPTLC-bioautography was performed to find the possible antibacterial fraction of essential oil (Figure 2).

However, results obtained indicated that phytopathogens strains were more sensitive than human pathogens after treatment with the tested essential oil. In particular, the Gram positive C. *michiganensis* subsp. *nebraskensis* resulted the most sensitivity, evidencing the lowest MIC value of 125 μg/mL. Gram negative bacteria showed resistance to *M. splendens* essential oil except for *P. syringae* pv. *syringae* that showed the lowest MIC value of 250 μg/mL. HPTLC-bioautography on *S. aureus* (Figure 2) exhibited two different areas corresponding to the fractions R_f_ 0.45–R_f_ 0.6 and R_f_ 0.7. The R_f_ 0.45–R_f_ 0.6 TLC area appeared as the most bioactive in terms of antibacterial activity and it was totally characterized by *trans*-nerolidol (100%). The fraction R_f_ 0.7 was mainly composed by β-cedren-9-one (87.34%). Data obtained suggested that *trans*-nerolidol is mainly responsible for the antibacterial activity of *M. splendens* essential oil that is in accordance with the results obtained by various authors. Simoes et al. [26] found that *trans*-nerolidol in combination with synthetic antibiotics ciprofloxacin, erythromycin, and gentamicin enhances their antibacterial activity against *S. aureus* and *E. coli.* Essential oils containing *trans*-nerolidol have been reported to inhibit the growth of quite a number of bacteria including *S. aureus* [27,28,29], *S. epidermidis* [30], *E. coli*, and *P. aeruginosa* [28,29]. Nerolidol (a mixture of *cis*- and *trans*-nerolidol) is also used as a food flavoring agent [27]. Considering β-cedren-9-one, the main component of R_f_ 0.7 fraction, few literature references are reported: to the best of our knowledge, this sesquiterpene is the main compound of *Artemisias persica* root essential oil [31], but no data are available for its antibacterial properties.

### 2.4. Antioxidant Activity

*M. splendens* essential oil exhibited antioxidant activity at higher concentration if compared to positive control. In fact, *M. splendens* essential oil showed a DPPH scavenging activity (IC_50_ = 43,537 ± 15 μg/mL) which is much lower than vitamin E (IC_50_ = 7.8 ± 0.5 μg/mL) (Table 4).

**Figure 3:**
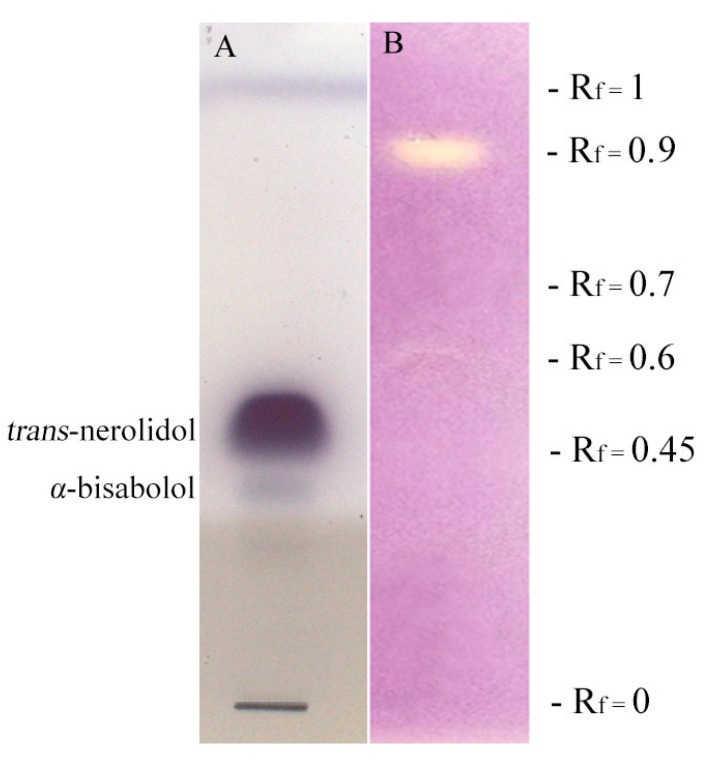
HPTLC bioautographic assay performed for DPPH activity of *M. splendens* essential oil. R_f_ composition is reported in Table 1. (**A**) HPTLC derivatized with vanillin-phosphoric acid reagent; (**B**) HPTLC derivatized with solution of DPPH radical.

The DPPH-HPTLC (1,1-diphenyl-2-picrylhydrazil—high performance thin layer chromatography) bioautographic assay (Figure 3) indicated that β-caryophyllene (36.23%), *trans*-γ-bisabolene (10.04%), *cis*-γ-bisabolene (8.33%), and *trans*-β-farnesene (7.81%) were the major compounds responsible for the lower scavenging activity at R_f_ 0.9 (Table 1).

These sesquiterpenes are present at low amount in the essential oil and that may be the reason for the low antioxidant activity. In addition, other explanation could be that antioxidant activity is reduced by some compounds which exhibits oxidant activity. This hypothesis seems to be confirmed by previous research related to the evaluation of cytotoxic activity of *Piper gaudichaudianum* Kunth essential oil, rich in *trans*-nerolidol, against *Saccharomyces cerevisiae*. The cytotoxic effect increased according to the absence of the superoxide dismutase (SOD), indicating that essential oil and *trans*-nerolidol developed reactive oxygen species (ROS). ROS production was confirmed by 2′,7′-dichlorofluorescein dictate (DCF-DA) test, on strains that do not produce the SOD enzyme. Cytotoxicity of *P. gaudichaudianum* essential oil and *trans*-nerolidol depends on ROS and DNA single strand breaks generated by presence of oxidative lesions [32]. With these premises, the high concentration (67.81%) of *trans*-nerolidol found in Ecuadorian *M. splendens* essential oil could explain the weak antioxidant activity in the DPPH assay.

## 3. Materials and Methods

### 3.1. Plant Material

The leaves of *M. splendens* were collected at the CIPCA (Centre for Research, Postgraduate and Conservation of the Amazon) of the Universidad Estatal Amazónica (UEA) (01°14′13″ S, 077°53′25″ W, 570 m) in April 2013, from a wild population in the Amazonian region of Napo, Ecuador. Species authentication was performed by Dr. David Neill. Voucher specimens were deposited at the Herbarium ECUAMZ of the UEA, in Ecuador (voucher specimen: David Neill 17351).

### 3.2. Isolation of Essential Oil

The essential oil was obtained from fresh leaves by hydrodistillation for 2 h in a stainless steel distiller equipped with a Clevenger-type apparatus. Essential oil yield (0.11%) was calculated on a moisture-free basis and determined as average of three distinct distillations. The oil was dried using anhydrous sodium sulfate and stored in sealed amber vials at 4 °C, for further analysis.

### 3.3. Chemicals

All chromatographic grade organic solvents, all reference standards and reagents used for GC analysis and for biological activities were purchased from Sigma-Aldrich (Milan, Italy), microbial culture media from Oxoid (Milan, Italy).

### 3.4. GC and GC-MS Analysis of the Essential Oil

The essential oil was chemically analyzed and the relative peak areas for individual compounds were averaged. For the analysis a ThermoQuest GC-Trace gas-chromatograph (ThermoQuest Italia, Rodano, Italy) equipped with an FID detector and a Varian FactorFour VF-5ms poly-5% phenyl-95%-dimethylsiloxane column (internal diameter, 0.25 mm; length, 30 m; film thickness, 0.15 µm) were used. The following conditions were adopted: injector temperature 300 °C, FID temperature 300 °C, carrier (Helium) flow rate 1 mL/min and split ratio 1:50. Oven temperature was as follow: from 55 to 100 °C at a rate of 1 °C/min, from 100 to 250 °C at a rate of 5 °C/min and then kept constant at 250 °C for 15 min. The volume injected was 1 µL, previously dissolved in CH_2_Cl_2_. The oil percentage composition was performed by the normalization method from the GC peak areas, without using correction factors. The chemical characterization of essential oil compounds was performed by a Varian GC-3800 gas chromatograph (Palo Alto, CA, USA) equipped with a Varian MS-4000 mass spectrometer (Palo Alto, CA, USA) using electron impact and hooked to the NIST (National Institute of Standards and Technology) library. The conditions and column were the same described for GC analysis. The mass spectroscopy conditions were as follows: ionization voltage, 70 eV; emission current, 10 µAmp; scan rate, 1 scan/s; mass range, 29–400 Da; trap temperature, 150 °C, transfer line temperature, 300 °C. The essential oil components were characterized by comparing their relative retention time (AI) and the MS fragmentation pattern with those of other known essential oils, with pure compounds and by matching the MS fragmentations patterns and retention indices with the above mentioned mass spectra libraries, and with those in the literature [11]. The Arithmetic Index of the components was determined adding a C8–C32 n-alkanes (Sigma-Aldrich) to the essential oil before injecting in the GC-MS equipment and analyzed under the same conditions reported above [3].

### 3.5. In Vitro Cytotoxic Activity

The cytotoxic activity was performed on A549 (human lung cancer cell line), MCF-7 (human breast cancer cell line), and HaCaT (human keratinocytes) purchased at “Istituto Zooprofilattico Sperimentale della Lombardia e dell’Emilia-Romagna” (Brescia, Italy) and it was expressed as the concentration of sample that inhibited 50% of cell growth (IC_50_). The cell lines were grown in Dulbecco’s modified Eagle’s medium supplemented with 10% fetal bovine serum (FBS), 2 mM L-glutamine and 100 U/mL penicillin/streptomycin. They were grown in 75 cm^2^ flasks in an air atmosphere characterized by 5% humidity and 95% CO_2_ at 37 °C, until 80% confluence was reached. Cytotoxic activity was determined by MTT colorimetric assay [33] as reflected by the activity of succinate dehydrogenase. Cells were seeded in 96-well plates at a density of 2 × 10^4^ cells/well in 200 µL DMEM (Dulbecco’s Modified Eagle Medium) complete medium; a period of 24 h was given for ensuring the cell attachment. Then the culture medium was replaced with 200 µL medium containing different concentrations (from 1 to 200 µg/mL) of *M. splendens* essential oil. In order to understand the role of α-bisabolol and *trans*-nerolidol in the cytotoxic activity, commercially available standards of those compounds were tested following the same methodology. Negative control was exposed to vehicle only, corresponding to a medium containing 2% FBS. The positive control used was doxorubicin. After 48 h, the culture medium was removed and washed with PBS (phosphate-buffered saline) twice. A lather of 20 μL of MTT (5 mg/mL in PBS) was added in each well and the plates were incubated for 4 h at 37 °C. The medium was removed and replaced with 100 μL of dimethylsulfoxide to dissolve the formazan crystals. The extent of MTT reduction was measured spectrophotometrically at 570 nm using a microplate reader.

### 3.6. Antibacterial Activity: HPTLC Bioautographic Assay

Bioautographic assay on a high performance thin layer chromatography (HPTLC) plate and the minimum inhibitory concentration (MIC) were performed in order to evaluate antibacterial activity of *M. splendens* essential oil [34]. Antibacterial activity was assessed against Gram negative *Escherichia coli* (ATCC 4350), *Pseudomonas aeruginosa* (ATCC 27853), *Agrobacterium tumefaciens* (DSM 30207), *Agrobacterium vitis* (DSM 6583), *Pseudomonas syringae* pv. *syringae* (DSM 10604) and Gram positive *Listeria grayi* (DSM 20601), *Staphylococcus aureus* (ATCC 29230), *Staphylococcus epidermidis* (ATCC 14990), *Enterococcus faecalis* (ATCC 29212), and *Clavibacter michiganensis* subsp. *nebraskensis* (DSM 20400).

The HPTLC bioautography was carried out on human pathogen *S. aureus*, with the aim of defining particular constituents of *M. splendens* essential oil that are able to determine the antibacterial activity. The bacterium choice was led because of its high performance features on HPTLC plate assay. The assay was performed as follows: 30 μL of a solution of *M. splendens* essential oil (110 mg/mL in ethanol) were applied on two separate HPTLC plates as 10 mm wide bands with Linomat V (Camag), while 10 μL of a methanol solution of *trans*-nerolidol (25 mg/mL) and 10 μL of a methanol solution of α-bisabolol (8 mg/mL) were applied on a third one on the same spot, as a 10 mm wide band. Then, all plates were eluted with a solvent solution containing toluene/ethyl acetate/petroleum ether (93/7/20). Finally, the solvent was completely dried maintaining plates for 30 min at room temperature: the first one was disposed in Petri dishes together with Nutrient Agar medium previously added with the bacterial inoculum (10^5^ CFU/50 mL) and 0.25% of a 2,3,5-triphenyl-tetrazolium chloride water solution (20 mg/mL), as a growth indicator. Incubation occurred overnight at 37 °C. Antibacterial compounds, responsible for possible antibacterial properties, looked like yellow spots against a red colored background. The second plate was used to remove active spots on silica and to extract these in methanol solutions that was directly analyzed by GC-MS, while the third one was sprayed with vanillin-phosphoric acid reagent to visualize *trans*-nerolidol and α-bisabolol as reference standards [35].

### 3.7. Determination of Minimum Inhibitory Concentration (MIC)

The antibacterial activity was determined as MIC and analyzed by the microdilution method using 96-well micro titer plates [36]. Bacterial cultures were incubated overnight at 37 °C, in Tryptic Soy Broth, while Gram negative *A. tumefaciens* and *A. vitis* in Nutrient Broth. One hundred microliters of sterile medium together with 100 μL of serial dilutions of *M. splendens* essential oil previously dissolved in ethanol (100 mg/mL), were pipetted into all the micro-wells. Serial dilutions were prepared in order to obtain concentration ranges from 27 μg/mL to 2000 µg/mL. One hundred μL of bacterial culture standardized to 2 × 10^7^ CFU/mL was added to the wells and incubated at 37 °C for 16 h and at 26 °C for 24 h, for human and phytopathogens, respectively. After the incubation period, 40 μL of water solution (20 mg/mL) of 2,3,5-triphenyl-tetrazolium chloride was added to each well and then incubated. Microbial growth was checked by a microplate reader at 615 nm, after 30 min of incubation. Chloramphenicol was used as a positive control [34].

### 3.8. Antioxidant Properties

Antioxidant activity was measured through 1,1-diphenyl-2-picrylhydrazil (DPPH) spectrophotometric assay (using UV-Vis spectroscopy) and DPPH-HPTLC bioautographic assay [34].

#### 3.8.1. Spectrophotometric DPPH Assay

Ten microliters of *M. splendens* essential oil was dissolved into 900 µL of ethanol, then serial dilutions were assessed in order to obtain different concentrations (0.8–6.67 × 10^−4^ μL/mL). An aliquot (2.9 mL) of the ethanol solution of DPPH (4 mg/100 mL) was added to the essential oil solution. After a 30 min incubation, in an orbital shaker at 200 rpm, in the dark at room temperature, the mixture was placed in an UV-Vis spectrophotometer and the absorbance was read in triplicate against a blank at 517 nm. The DPPH inhibition in percent was determined by the following formula: IDPPH% = [1 − (A1/A2)] × 100. Where A1 was the DPPH absorbance with the essential oil and A2 without the essential oil. Vitamin E was used as positive control, according to its well-known antioxidant properties [37]. Essential oil antioxidant activity was expressed as IC_50_ (concentration providing DPPH 50% inhibition), calculated from inhibition curves obtained by plotting inhibition percentage against essential oil concentration. All experiments were assessed in triplicate and values were reported as mean ± SD (Standard Deviation).

#### 3.8.2. DPPH-HPTLC Bioautography

DPPH-HPTLC-bioautography was assessed to determine the active compounds of the essential oil, responsible for possible radical scavenging activity. Twelve microliters of an ethanol solution of *M. splendens* essential oil (110 mg/mL) were applied twice to a silica gel HPTLC plate as 10 mm wide bands with Linomat V. Then, spots were eluted with a solvent solution (toluene/ethyl acetate/petroleum ether 93/7/20) in a chromatographic chamber. After development, the first chromatogram was sprayed with the DPPH ethanol solution (20 mg/100 mL) to detect possible antioxidant fractions. The active compounds appeared as yellow areas on a violet background. Isolation and identification of antioxidant compounds were carried out removing the TLC areas in the second chromatogram at R_f_ corresponding to positive spots and then extracting them with methanol. The solutions were analyzed by GC-MS. The third plate obtained from HPTLC bioautographic assay was used to show *trans*-nerolidol and α-bisabolol as reference standards.

### 3.9. Statistical Analysis

The experiments were performed in triplicate. IC_50_ values of anti-proliferative and antioxidant activities were assessed by logarithmic regression curves with 95% confident limits. Relative standard deviations and statistical significance (Student’s *t* test; *p* ≤ 0.05) were calculated using software STATISTICA 6.0 (StatSoft Italia srl, Vigonza, Italy).

## 4. Conclusions

The present study characterized the chemical profile of *M. splendens* essential oil from Ecuador for the first time and investigated its cytotoxic, antibacterial, and antioxidant properties. The essential oil of this plant revealed a good potential against the MCF-7 cell line and suggested that further deeper investigations of in vitro and in vivo anti-cancer studies are needed. *M. splendens* essential oil showed negligible antioxidant activity and low antibacterial effects against Gram positive and negative human pathogens, and moderate activity against phytopathogen strains. In addition, it was found that the chemical characterization of *M. splendens* essential oil from Amazonian Ecuador differs qualitatively and quantitatively from essential oils obtained by plants obtained of the same species but coming from different regions. Moreover, *M. splendens* essential oil, as a source of *trans*-nerolidol, should have pharmacological applications because of its enhancing properties on improving skin absorption of a drug with low lipophilic features [38].

## Figures and Tables

**Table 1:** Chemical composition of *M. splendens* essential oil and its characterization in term of active antioxidant and antibacterial compounds isolated respectively from DPPH-HPTLC (1,1-diphenyl-2-picrylhydrazil-high performance thin layer chromatography) and HPTLC bioautography assays.

					Antioxi. F. ^5^		Antibact. F. ^6^	
No.	% Area ^1^	Component ^2^	AI Exp ^3^	AI Lit ^4^	R_f_ = 0.9	R_f_ = 0.45–0.6		R_f_ = 0.7
1	2.08 ± 0.10	α-pinene	928	939				
2	0.11 ± 0.01	β-pinene	972	979				
3	0.15 ± 0.02	α-cubebene	1350	1351	1.39			
4	0.51 ± 0.03	α-copaene	1375	1377	4.21			
5	0.11 ± 0.02	β-elemene	1387	1391	4.39			
6	4.21 ± 0.15	β-caryophyllene	1409	1419	36.23			
7	0.31 ± 0.02	*trans*-α-bergamotene	1430	1435	3.15			
8	0.45 ± 0.03	α-caryophyllene	1449	1455	3.94			
9	0.87 ± 0.06	*trans*-β-farnesene	1453	1457	7.81			
10	0.65 ± 0.04	germacrene D	1475	1485	4.62			
11	0.10 ± 0.01	*cis*-β-guaiene	1485	1493	1.54			
12	0.17 ± 0.02	viridiflorene	1490	1497	1.43			
13	0.11 ± 0.01	α-muurolene	1497	1499	1.27			
14	0.59 ± 0.03	α-bisabolene	1503	1507	1.24			
15	0.24 ± 0.02	*cis*-γ-bisabolene	1506	1515	8.33			
16	0.53 ± 0.04	δ-cadinene	1513	1523	4.93			
17	0.16 ± 0.01	*trans*-calamenene	1518	1529	1.66			
18	1.03 ± 0.09	*trans*-γ-bisabolene	1523	1531	10.04			
19	67.81 ± 2.10	*trans*-nerolidol	1562	1563		100		
20	0.15 ± 0.01	caryophyllene oxide	1580	1583				
21	Traces	β-cedren-9-one	1630	1631				87.34
22	17.51 ± 1.01	α-bisabolol	1690	1686				
Total identified	97.84							

^1^ Relative peak areas ± SEM (standard error media), calculated by GC-FID; ^2^ Components are listed in order of elution and their nomenclature is in accordance of the NIST (National Institute of Standards and Technology) library; ^3^ AI exp: arithmetic indices calculated on a Varian VF-5ms column; ^4^ AI lit: arithmetic indices [11]; ^5^ Antioxi. F.: fraction of compounds responsible for antioxidant activity. ^6^ Antibact. F.: fraction of compounds responsible for antibacterial activity.

**Table 2:** Cytotoxic activity of *M. splendens* EO, α-bisabolol and *trans*-nerolidol on three cell lines after 48 h.

	Cell Line (IC_50_ µg/mL) ^1^		
	A549 ^2^	MCF-7 ^3^	HaCaT ^4^
*M. splendens* EO	100.99 ± 2.32 ^6d^	5.59 ± 0.13 ^6c^	21.58 ± 1.26 ^6c^
α-bisabolol	27.63 ± 2.01 ^6b^	1.24 ± 0.03 ^6a^	10.15 ± 0.35 ^6b^
*trans*-nerolidol	54.28 ± 2.39 ^6c^	40.97 ± 5.07 ^6d^	27.76 ± 2.76 ^6d^
doxorubicin ^5^	0.90 ± 0.01 ^6a^	2.10 ± 0.42 ^6b^	0.40 ± 0.01 ^6a^

^1^ IC_50_: compound concentrations that affords a 50% cell growth decrease after 48 h. IC_50_ are the averages of triplicate experiments and represented as mean ± standard deviation; ^2^ Adenocarcinoma cell line; ^3^ Breast adenocarcinoma cell line; ^4^ Keratinocytes cell line; ^5^ Doxorubicin was used as positive control; ^6^ Data are presented as mean ± SD, *n* = 3. Means in each column followed by different letter are significantly different (*p* < 0.05).

**Table 3:** Antibacterial activity of *M. splendens* essential oil expressed as MIC (Minimum Inhibitory Concentration) (μg/mL).

	Strain	*M. splendens* EO ^1^	Antibiotic ^2^
Gram negative			
*Agrobacterium tumefaciens*	DSM 30207	500 ^3c^	6.25 ^3c^
*Agrobacterium vitis*	DSM 6583	2000 ^3e^	1.56 ^3a^
*Pseudomonas syringae* pv. *syringae*	DSM 10604	250 ^3b^	3.12 ^3b^
*Escherichia coli*	ATCC 4350	>2000 ^3f^	25.00 ^3d^
*Pseudomonas aeruginosa*	ATCC 27853	>2000 ^3f^	6.25 ^3c^
**Gram positive**			
*Clavibacter michiganensis* subsp. *nebraskensis*	DSM 20400	125 ^3a^	6.25 ^3c^
*Enterococcus faecalis*	ATCC 29212	2000 ^3e^	3.12 ^3b^
*Listeria grayi*	DSM 20601	1000 ^3d^	5.00 ^3c^
*Staphylococcus aureus*	ATCC 29230	1000 ^3d^	3.12 ^3b^
*Staphylococcus epidermidis*	ATCC 14990	1000 ^3d^	3.12 ^3b^

^1^ EO: Essential Oil; ^2^ Chloramphenicol was used as positive control; ^3^ Means in each column followed by different letters are significantly different (*p* < 0.05).

**Table 4:** Antioxidant activity of *M. splendens* essential oil compared with the standard (vitamin E).

Sample	IC_50_ (μg/mL) ^1^
*M. splendens* EO	43,537.00 ± 15 ^3b^
vitamin E ^2^	7.8 ± 0.5 ^3a^

^1^ IC_50_: DPPH scavenging activity of EO was expressed as IC_50_ (µg/mL) value; ^2^ Vitamin E was used as positive control; ^3^ Data are presented as mean ± SD, *n* = 3. Means in each column followed by different letter are significantly different (*p* < 0.05).
